# The Coming Unification of the Medical Profession

**Published:** 1902-05

**Authors:** 


A Monthly Review of Medicine and Surgery.

*EDITOR:*

WILLIAM WARREN POTTER, M. D.

All communications, whether of a literary or business nature, books for review and exchanges should be addressed to the editor: 284 Franklin Street, Buffalo, N. Y.

Vol. XLI.—LVII. MAY, 1902. No. 10.

### "The Coming Unification of the Medical Profession."

AT THE opening of the Physicians' Club at Dayton, O., which occurred March 20, 1902, the dedicatory address was delivered by Professor Charles A. L. Reed, of Cincinnati, the distinguished ex-president of the American Medical Association. Dr. Reed chose for the subject of his remarks The Coming Unification of the Medical Profession,—a subject that is just now paramount in the minds of the leaders of professional thought in the state of New York.

In the March edition of this journal, announcement was made of the appointment of committees of conference representing each of the two state medical societies, the object of which was stated to be to devise a plan for the union of the two bodies, and thus to put an end to the schism which has existed in the Empire state for nearly twenty years. These committees have had several meetings already, and it is believed substantial progress has been made toward the much desired end in view; at all events, there is no reason why such should not be the case, for the way thereunto has already been smoothly paved by the action of the American Medical Association during its meeting at St. Paul last year.

It will be remembered that at that time, under the inspiration of the president, Dr. Reed, a complete reorganisation was secured and a new constitution and by-laws adopted, which completely swept away all the elements in the old organisation that had caused differences of opinion between the National body and the medical society of the state of New York. The association announces in its second article that its object "shall be to federate into one compact organisation the medical profession of the United States . . . . and of representing to the world the practical accomplishments of scientific medicine." This would seem to be broad enough and the door to be open wide enough to permit the entrance of every legally qualified physician in the United States.

In the by-laws as then adopted, Chapter XV. says: " . . . . and provided, further, that nothing in these by-laws shall be construed to repeal the rules of the Association governing the relation of members to each other and to the Association." This is the only section that possibly might be construed to refer to the code. It is a principle of law that only such exemptions or exceptions as are specifically named in a statute remain, while all others are repealed. Therefore, it will readily be seen that whatever of the code is retained, is simply recognised as "rules" and has not the force of law. The rules embraced in the code of ethics which are repealed by the foregoing proviso are those pertaining to the relations of members of the association. The rules that were not thus repealed now, alone, constitute the code of ethics, if it be entitled to such name, and the publication by any association or individual of any document purporting to be the code of ethics of the American Medical Association, the provisions of which are not in accordance with the foregoing facts, would be in derogation of the rights of individual members of the American Medical Association, and would subject that incorporated body to proceedings in injunction before any court of competent jurisdiction.

This legislation, or action, of the American Medical Association removes the last ethical barrier between it and the Medical Society of the State of New York. The code controversy, therefore, is at an end and should be buried alongside of the ancient and respectable document which gave rise to all the difficulty, and which never again will be printed except as a historic curio.

It will be observed, therefore, that the American Medical Association has done all that it can do,—indeed, all that it could be expected to do,—to bring together the contending factions that were rent in twain by its own action in 1882, in rejecting the delegates from New York, because the society they represented had adopted a modified code of ethics. Nothing would seem to remain to reëstablish harmony but to arrange "a few purely trivial details," which relate rather to the commercial than to the professional side of the question.

In his Dayton address Dr. Reed makes many observations that are very pertinent to this general subject, from which we quote the following paragraphs taken from the *New York Medical Journal* of April 19, 1902. After reviewing the purposes of the Physicians' Club which he was addressing, and which were stated to be to gather within its folds the legally qualified physicians of Dayton, without reference to "schools" or "sects" to discuss such things as are of importance to the public welfare, and of interest to themselves professionally, politically, and commercially, he proceeded to say:

EXCERPTS FROM DR. REED'S ADDRESS.

I congratulate you, in the first place, upon having effected this organisation upon the broad basis of good citizenship. You have recognised the primacy of the law, not only by conforming to its provisions in your individual relations to the State, but tonight you have gone further, by bowing with loyalty to that sentiment that makes law possible, this sentiment, more important in a democracy than the law itself,—the sentiment that, while underlying the law, rises above it,—must be recognised in all walks of life, in all ranks of society, as a chief safeguard of the Republic. That profession, craft, or calling which, in its organised capacity, declines to recognise that which the law recognises lends itself to the propagation of a sentiment that is inimical to law itself.

The state, by authority of the governed, has authority, not only to protect society against imposition, but to secure to it the best developments in any department of science. In the exercise of this authority the various states of the Union with unanimity have declared that one who shall assume responsibility, professionally, for the treatment, care, and conduct of the sick and injured shall have a certain knowledge of the fundamental branches of medicine, which includes anatomy, physiology, chemistry, hygiene, obstetrics and surgery; and the states have, with almost equal unanimity assumed, in effect, that physicians who are competently educated in these departments may be left either to themselves or to the supervision of their sectarian associates in matters of therapeutics. This has been and is the wise middle ground, the fruits of which are obvious all over the country—are obvious here tonight—where, with a complete understanding upon the fundamental branches, and with an abandonment to the spirit of truth, an open, frank, free, and understandable discussion may be had upon all correlated subjects of science. But the essential, the paramount question, is that of the fundamental studies without which, as a basis, no system of medicine, whatever its sectarian title, can lay the slightest claim to being scientific. . . . . . . . . . .

As a matter of fact, the regime that we are inaugurating so auspiciously in Ohio tonight, and the principles of organisation such as you have here adopted, have become rather extensively established in the American medical profession. It began as long ago as 1876, when the physicians of California assembled without reference to denominational predilections, and secured the adoption of the first effective law for the regulation of the practice of medicine anywhere in the United States. It is true that those conferences, called for temporary purposes, did not assume the form of permanent organisation for social and scientific purposes, such as you have here established, but the principle of reciprocal recognition and coöperation was then and there promulgated. The same steps were taken during the succeeding few years in Illinois, Alabama, and Colorado. In each instance the movements resulted in the establishment of state licensing boards, composed of representatives of the different "schools" or "sects," who consulted, yes, actually consulted, not upon the trivial question of a dose of medicine, but upon the vastly more vital question of the qualifications of those who were to practise the healing art. Thus it happened that, under the influence of the state, instigated by the profession itself, the lines of demarcation between the contending denominations of physicians were subjected to the gradual but sure process of effacement. From these beginnings the process has spread all over the country, and it has been accomplished, with but a single exception, with that smoothness characteristic of evolutional growth.

The only exception was that of the State of New York, in which the physicians of the dominant and controlling school of practice, as a result of doing precisely what had been done without protest in the other states,—as a penalty for doing precisely what you are doing here tonight,—was subjected to the penalty of excommunication by its national organisation. This action has stood for twenty years as the one conspicuous stain upon the escutcheon of the American medical profession. I am happy to state that, under the broader knowledge of today, this wrong is about to be remedied. I feel that I am violating no confidence when I state here tonight that committees, representing the affiliated body of the national organisation on the one hand and the excommunicated state organisation on the other, each actuated by the *Zeitgeist* of the twentieth century, are arranging a few purely trivial details with the object of reëstablishing the complete unity of the national profession. I may go a step further and say to you, in all confidence, that the great sentiment of the American Medical Association touching this unfortunate incident is such that it awaits with impatience the successful completion of these negotiations. You may, therefore, look forward with confidence to the meeting to be held in Saratoga in June as the date which shall mark the close of that period in our national profession when a reputable physician shall be denied recognition and fellowship because he exercises the most fundamental prerogatives of individual liberty.

It is, indeed, the dawn of a better epoch, when members of a learned profession, and a profession with growing liberality, maintain an open tribunal before which may be represented any scientific truth. It bespeaks an era when the professional mind, untrammeled by dictum or authority, shall assume a judicial poise. It is an era in which the chief point of pride shall be, not to maintain a preconception for the sake of personal achievement, but gladly to yield it in the light of demonstrated truth. With such a spirit dominating our profession, and with the unwritten code of the gentleman controlling the personal conduct and the professional relations of its members, we may feel that we are in the way of fulfilling, in the highest degree, our functions as the conservators of the public welfare. In conclusion and just as the sun is breaking in richest effulgence upon this new day, I can only congratulate you for having thus earnestly and auspiciously demonstrated what is meant by that increasing purpose that runs through the ages and by those thoughts of men that broaden with the procession of the suns.

The action of the Dayton Physicians' Club is on similar lines taken by the Buffalo Ophthalmological Club recently organised in this city, the details of which will be found on another page in this issue. The only difference is that, in Dayton, a declaration was made in the preliminary notice sent out; whereas, at Buffalo, homeopathists, state association men and state society men, and physicians that did not belong to any of these organisations, united in forming a club with the object of discussing scientific medicine. The first meeting for this purpose was held at the home of Dr. F. Park Lewis, a distinguished representative of the homeopathic medical profession, and a scientic contribution from whom will be found in another part of this magazine. Among others who attended this meeting and participated in its proceedings was Dr. A. A. Hubbell, president of the New York State Medical Association for the current year. Under these conditions, which are not singular but are occurring here and there all over the country, would it not seem that the time has arrived when every legally qualified physician in good standing with his professional confrères might accept the invitation of, and be admitted into that distinguished representative body of the medical profession,—the American Medical Association?

